# The Cytoplasmic Tail Domain of Epstein-Barr Virus gH Regulates Membrane Fusion Activity through Altering gH Binding to gp42 and Epithelial Cell Attachment

**DOI:** 10.1128/mBio.01871-16

**Published:** 2016-11-15

**Authors:** Jia Chen, Theodore S. Jardetzky, Richard Longnecker

**Affiliations:** a Department of Microbiology and Immunology, The Feinberg School of Medicine, Northwestern University, Chicago, Illinois, USA; b Department of Structural Biology, Stanford University School of Medicine, Stanford, California, USA

## Abstract

Epstein-Barr virus (EBV) is associated with infectious mononucleosis and a variety of cancers as well as lymphoproliferative disorders in immunocompromised patients. EBV mediates viral entry into epithelial and B cells using fusion machinery composed of four glycoproteins: gB, the gH/gL complex, and gp42. gB and gH/gL are required for both epithelial and B cell fusion. The specific role of gH/gL in fusion has been the most elusive among the required herpesvirus entry glycoproteins. Previous mutational studies have focused on the ectodomain of EBV gH and not on the gH cytoplasmic tail domain (CTD). In this study, we chose to examine the function of the gH CTD by making serial gH truncation mutants as well as amino acid substitution mutants to determine the importance of the gH CTD in epithelial and B cell fusion. Truncation of 8 amino acids (aa 698 to 706) of the gH CTD resulted in diminished fusion activity using a virus-free syncytium formation assay and fusion assay. The importance of the amino acid composition of the gH CTD was also investigated by amino acid substitutions that altered the hydrophobicity or hydrophilicity of the CTD. These mutations also resulted in diminished fusion activity. Interestingly, some of the gH CTD truncation mutants and hydrophilic tail substitution mutants lost the ability to bind to gp42 and epithelial cells. In summary, our studies indicate that the gH CTD is an important functional domain.

## IMPORTANCE

Infection with Epstein-Barr virus (EBV) causes diseases ranging from the fairly benign infectious mononucleosis to life-threatening cancer. Entry into target cells is the first step for viral infection and is important for EBV to cause disease. Understanding the EBV entry mechanism is useful for the development of infection inhibitors and developing EBV vaccine approaches. Epithelial and B cells are the main target cells for EBV infection. The essential glycoproteins for EBV entry include gB, gH/gL, and gp42. We characterized the function of the EBV gH C-terminal cytoplasmic tail domain (CTD) in fusion using a panel of gH CTD truncation or substitution mutants. We found that the gH CTD regulates fusion by altering gp42 and epithelial cell attachment. Our studies may lead to a better understanding of EBV fusion and entry, which may result in novel therapies that target the EBV entry step.

## INTRODUCTION

Epstein-Barr virus (EBV) is a human pathogen that typically results in asymptomatic infection in preadolescent children but can result in infectious mononucleosis in adolescents and adults. Primary infection with EBV is thought to initiate in epithelial cells of the oral pharynx. Transmission by sexual, transfusion, and transplantation routes has also been reported for EBV. Most important for EBV persistence in the human host is the targeting of B cells by EBV, where the virus establishes a latent infection. It is from these latently infected cells that virus lytic replication initiates, providing infectious virus for the infection of naive hosts (1).

EBV is an enveloped double-stranded DNA virus that enters target cells through the fusion of the virion envelope with a host cell membrane. Four viral-membrane-associated proteins have been determined as the minimal glycoproteins for B cell entry using virus-free cell-cell fusion. These are glycoprotein 42 (gp42), gH, gL, and gB. The requirements for fusion of epithelial and B cells differ but include the core fusion machinery gH/gL and gB (2). gp42 is required only for B cell fusion but inhibits epithelial cell fusion, acting as a tropism switch by directing the entry of EBV into B cells or epithelial cells (3). The crystal structure of the ectodomain of EBV gB and gH/gL has been solved (4, 5), and it is similar to those of other herpesvirus gBs and gH/gLs (6–9). The secreted EBV gB ectodomain forms 16-nm-long spike-like trimers, structurally homologous to the postfusion trimers of the fusion protein G of vesicular stomatitis virus (VSV) (4). The heterodimeric complex of gH/gL was identified as an elongated rod-like shape that differs from the “boot-like” structure of herpes simplex virus (HSV) gH/gL (5). More recently, we determined the electron microscopy (EM) structure of the B cell triggering complex comprised of gH/gL, gp42, and HLA class II that is required for the infection of B cells by EBV (10). This structure provided a unique opportunity to further understand herpesvirus-induced membrane fusion.

The specific role that gH/gL plays in fusion has been the most elusive among the required herpesvirus entry glycoproteins. From our studies, including the structural studies described above, as well as the work of others studying the role of gH/gL in herpesvirus entry, gH/gL appears to function as a bridge between receptor binding and activation of gB to mediate fusion (11). EBV gH is a transmembrane protein containing 706 amino acids (aa). The transmembrane domain (TMD) of gH constitutes amino acids 679 to 698. Thus, only 8 amino acids are contained in the gH cyoplasmic tail domain (CTD). The X-ray crystallographic structure of the EBV gH/gL ectodomain indicates that there is a linear 4-domain arrangement (domains I to IV) forming a flat elongated shape with individual domains arranged in tandem along the length of the molecule. The hydrophobic interface of D-I and D-II is linked by an α-helix and reflects that gH/gL domains may undergo dynamic rearrangement (5). There is also a large groove between D-I and D-II adjacent to the gH/gL KGD motif located on the surface of D-II of gH/gL (5). Our mutagenesis studies have shown that this groove participates in both B and epithelial cell fusion and indicate that flexibility between D-I and D-II in gH/gL is required for epithelial cell but not B cell fusion (12).

Previous mutational studies have been focused on studying the ectodomain of EBV gH/gL (10–14) and not on the gH/gL CTD. Recent data from our laboratory indicate that soluble gH/gL lacking the gH TMD and the CTD does not function in B cell fusion and instead inhibits B cell fusion (15). Studies with herpes simplex virus 1 (HSV-1) gH/gL TMD as well as the CTD prior to our studies suggested an important role of the gH CTD in membrane fusion. Similarly to our observations here, HSV-1 gH/gL mutants that lack the authentic TMD or CTD are unable to mediate cell-cell fusion when coexpressed with gB (16). In addition, a conserved glycine residue 812 mutant within the HSV-1 gH TMD or a truncation at aa 832 coupled with the point mutation V831A within the CTD of gH reduces fusion function (16, 17). The addition of a linker insertion at residue 824 within the short HSV-1 gH CTD completely abrogated fusion (18). A mutant membrane-bound form of gD (gDgpi) lacking the transmembrane and cytoplasmic tail of gD, which were replaced with a glycophosphatidylinositol linkage (gpi) or chimeric gD molecule in which the membrane anchor and cytoplasmic tail domains were replaced with analogous regions from the human CD8 molecule, promoted fusion to near-wild-type (WT) levels (19, 20). However, similar HSV-1 mutants with gH and gB (gHgpi and gBgpi) were unable to promote fusion. These data together highlight that the TMD and CTD are important functional domains for gH and gB.

Our previous work investigating the role of the EBV gB cytoplasmic tail domain in cell fusion led to the identification of regions that are important for cell fusion and regions in the EBV gB ectodomain that undergo conformational changes required for fusion function. To further elucidate whether the EBV gH CTD is important for gH function, we generated a series of gH mutants in which the gH CTD was truncated or contained amino acid substitutions. In this study, we report that the regulation of fusion activity by the CTD of gH depends on the length of the domain and the specific amino acid composition of the gH CTD.

## RESULTS

### Characterization of the cell surface expression, cell-cell fusion, and gp42 binding of gH cytoplasmic tail deletion mutants.

**FIG 1:**
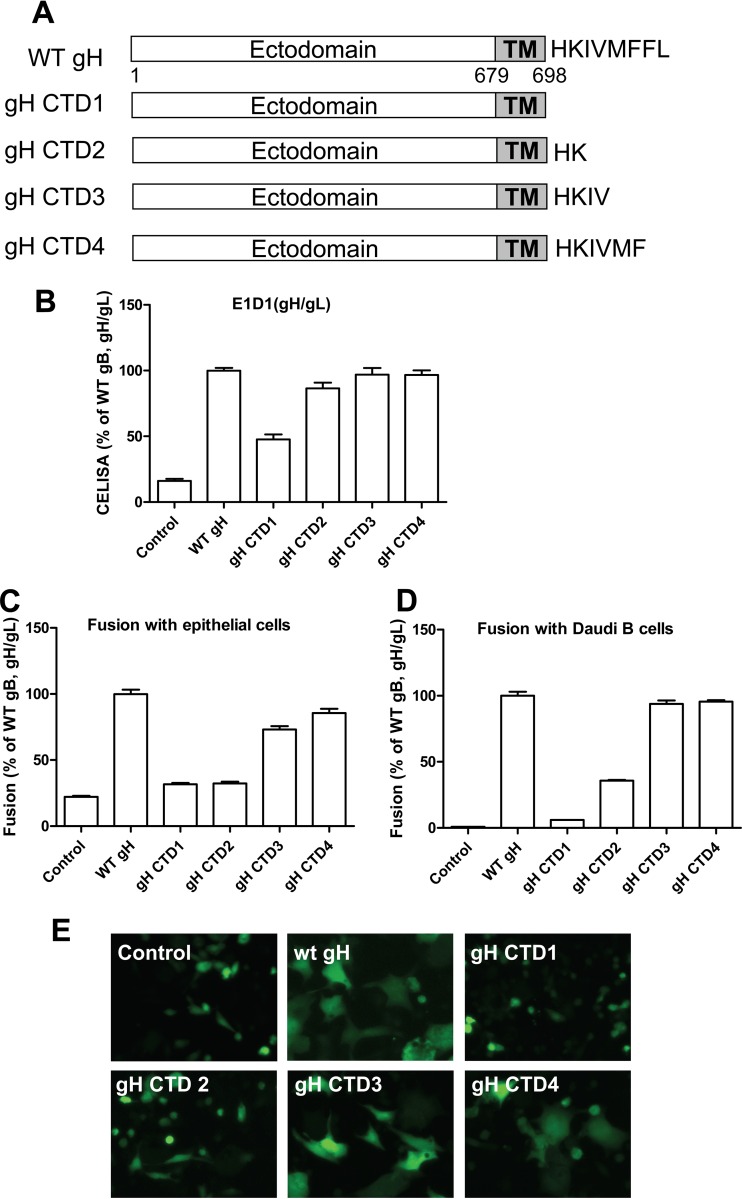
The EBV gH/gL CTD truncation mutants alter fusion activity and cell surface expression. (A) Schematic of gH CTD mutants. (B to D) CHO-K1 cells were transiently transfected with T7 luciferase plasmid alone with vector plasmid (control), T7 luciferase plasmid, or EBV gB or gL, together with gH or gH CTD mutants in the absence (B and C) or presence (D) of gp42. Twenty-four hours posttransfection, transfected CHO-K1 cells were overlaid with epithelial (C) or Daudi B (D) cells expressing T7 polymerase, and luciferase activity was monitored 24 h after overlay and normalized to cells with the WT gH/gL value set to 100%. The data are the mean plus standard error of the mean from three independent experiments. (B) Expression of gH/gL. Twenty-four hours posttransfection, 4 × 10^4^ cells were seeded into 96-well plates in triplicate. CELISA was performed with anti-gH/gL MAb E1D1. (E) CHO-K1 cells were transfected with green fluorescent protein plasmid alone with vector plasmid only (control), green fluorescent protein plasmid, or EBV gL or gB with different gH mutants as indicated and overlaid with HEK 293 cells. Syncytium formation was visualized and captured with a Leica fluorescence microscope.

**FIG 2:**
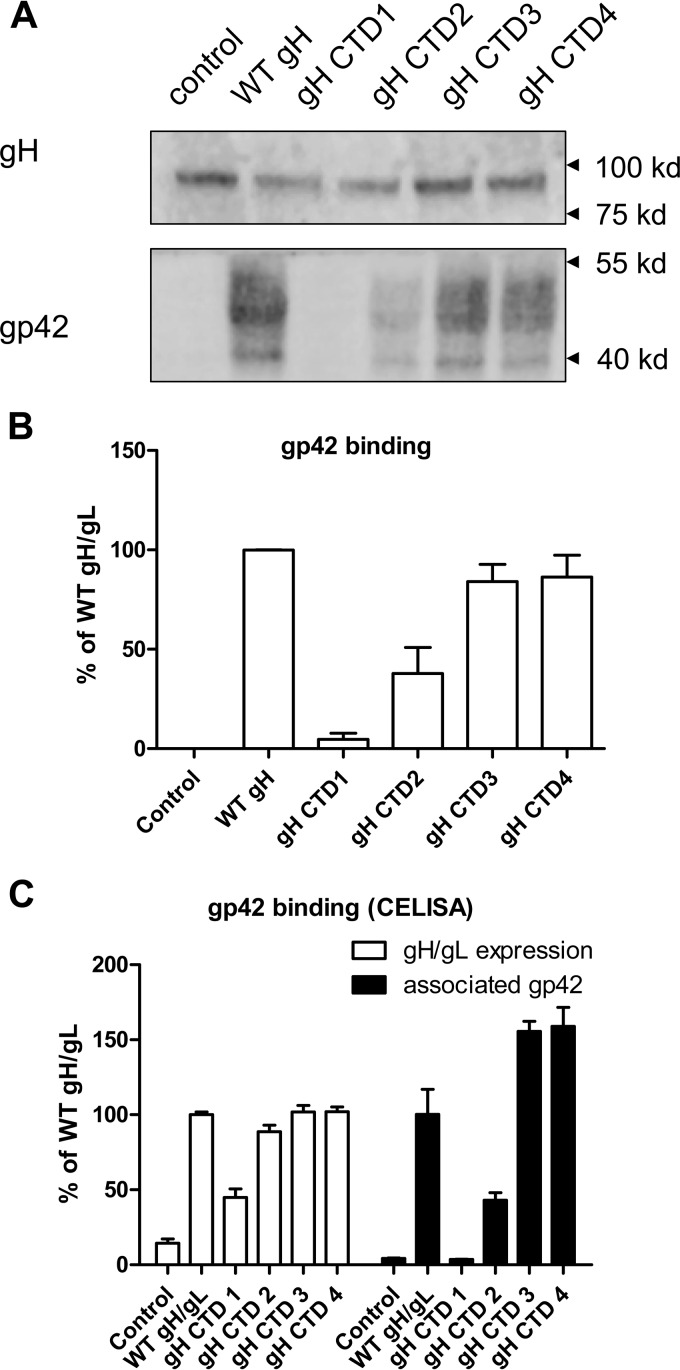
gH/gL CTD truncation mutants have decreased association with gp42. (A) CHO-K1 cells seeded in six-well plates were transfected with control, WT gH/gL, or gH/gL mutants as indicated. A gp42 binding assay was performed to detect the Flag-tagged gp42 that bound to the transfected cells. Anti-gH was used as a loading control. (B) The relative level of gp42 binding was quantified and was normalized to the level of gH by using Image Studio software. The binding of WT gH/gL to gp42 was set at 100%. Data are means plus standard errors of the means for three independent experiments. (C) CHO-K1 cells seeded in six-well plates were transfected with control, WT gH/gL, or gH/gL mutants as indicated. Transfected cells were seeded in a 96-well plate in triplicate posttransfection. After 24 h, cells were washed twice with ice-cold PBS and incubated with soluble Flag-gp42 for 1 h at 4°C. The cells were then washed with ice-cold PBS four times, and the gH/gL surface expression and associated gp42 were determined by CELISA using antibodies E1D1 (gH/gL expression) and PB1112 (associated gp42).

Previous studies of the HSV gH tail and the varicella-zoster virus (VZV) gH tail found that truncation of the gH cytoplasmic tail domain (CTD) alters fusion activity (17, 21, 22). Deletion, replacement, or insertion mutations of the transmembrane domain (TMD) and gH CTD inhibit the fusion activity of HSV gH without altering gH/gL cell surface expression (16). These results indicate that the CTD of gH can positively regulate fusion activity. In contrast, VZV gH CTD mutants have an increase in cell fusion activity with an 8-amino-acid (aa 834 to 841) truncation (21). EBV gH has an 8-amino-acid CTD which is shorter than the 14-amino-acid HSV gH CTD and 18-amino-acid VZV gH CTD. To evaluate the role of the EBV gH CTD in fusion activity, we generated serial gH truncation mutants as shown in Fig. 1A and characterized each of the mutants for cell surface expression and fusion activity. We found that the deletion of the entire gH CTD results in an approximately 50% reduction in cell surface expression (Fig. 1B) compared to wild-type (WT) gH/gL, indicating that the gH CTD is important for cell surface expression. In contrast to the gH CTD1 mutant with the entire cytoplasmic gH tail truncated, all the other EBV gH CTD truncation mutants had cell surface expression similar to that of WT gH (Fig. 1B). Using the virus-free cell-cell fusion assay, we tested the effect of these mutations on fusion with both epithelial and B cells. Consistent with the results that soluble EBV gH cannot function in fusion and the observation that deletion, replacement, or insertion mutants inhibit the fusion activity of HSV gH (15, 16), we found that the EBV gH CTD is important for fusion activity (Fig. 1C and D). The gH CTD1 mutant with the entire cytoplasmic tail truncated had a 50% decrease in cell surface expression and lacked fusion activity for both B cell and epithelial cell fusion. The gH CTD2 mutation had normal cell surface expression (Fig. 1B) but very low fusion activity for epithelial cells and about 25% of WT fusion activity for B cells (Fig. 1C and D). The gH CTD3 and gH CTD4 mutants had WT levels for B cell fusion (Fig. 1D) and a small decrease in epithelial cell fusion activity for gH CTD3 (Fig. 1C), indicating that the last 4 amino acids are not important for fusion activity. Using a syncytium formation assay, we observed very similar results in regard to fusion function of the mutants (Fig. 1E). We previously reported that the CTD of gB also regulates fusion activity (23, 24). Since gp42 binding to gH/gL is essential for B cell fusion, we investigated the binding of gp42 to the gH CTD mutants using a monolayer cell binding assay as described in Materials and Methods. The gH CTD1 deletion mutant was nearly negative for B cell fusion. Interestingly, we found that both the gH CTD1 and gH CTD2 mutants had a dramatic decrease for gp42 binding as shown in Fig. 2A. Quantification of gp42 binding (Fig. 2B) correlates with the B cell fusion activity (Fig. 1D), indicating that that the gH CTD may influence gH/gL binding to gp42. The gp42 binding data were further confirmed using a cell enzyme-linked immunosorbent assay (CELISA) as described in Materials and Methods (Fig. 2C). These gp42 results parallel the Western blotting results, further demonstrating that the gH CTD1 and gH CTD2 mutants had diminished gp42 binding (Fig. 2A to C).

### Characterization of gH CTD mutants with hydrophobic, hydrophilic, and random amino acid changes in the gH CTD for cell surface expression, cell-cell fusion, and gp42 binding.

**FIG 3:**
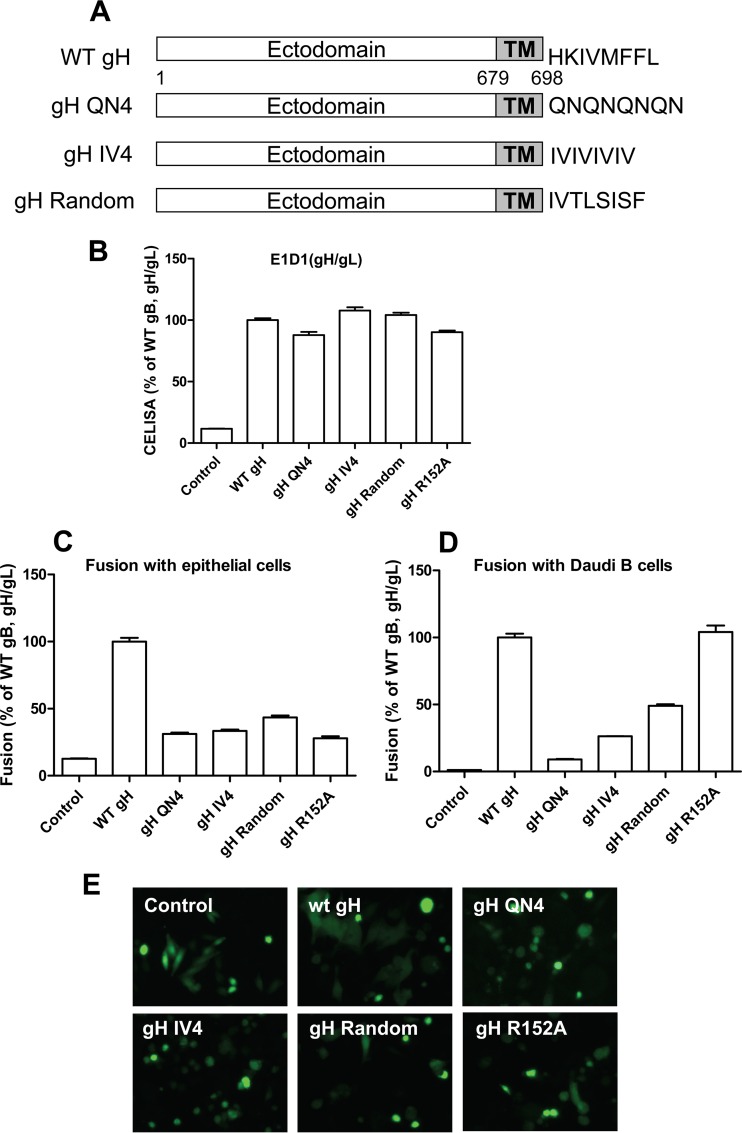
The EBV gH/gL CTD amino acid composition is important for fusion activity. (A) Schematic of gH CTD mutants. (B to D) CHO-K1 cells were transiently transfected with T7 luciferase plasmid alone with vector plasmid (control), T7 luciferase plasmid, or EBV gB or gL, together with gH or gH CTD mutants or gH R152A in the absence (B and C) or presence (D) of gp42. Twenty-four hours posttransfection, transfected CHO-K1 cells were overlaid with epithelial (C) or Daudi B (D) cells expressing T7 polymerase, and luciferase activity was monitored 24 h after overlay and normalized to cells with the WT gH/gL value set to 100%. The data are the mean plus standard error of the mean from three independent experiments. (B) Expression of gH/gL. Twenty-four hours posttransfection, 4 × 10^4^ cells were seeded into 96-well plates in triplicate. CELISA was performed with anti-gH/gL MAb E1D1. (E) CHO-K1 cells were transfected with green fluorescent protein plasmid alone with vector plasmid only (control), green fluorescent protein plasmid, or EBV gL or gB with different gH mutants as indicated and overlaid with HEK 293 cells. Syncytium formation was visualized and captured with a Leica fluorescence microscope.

**FIG 4:**
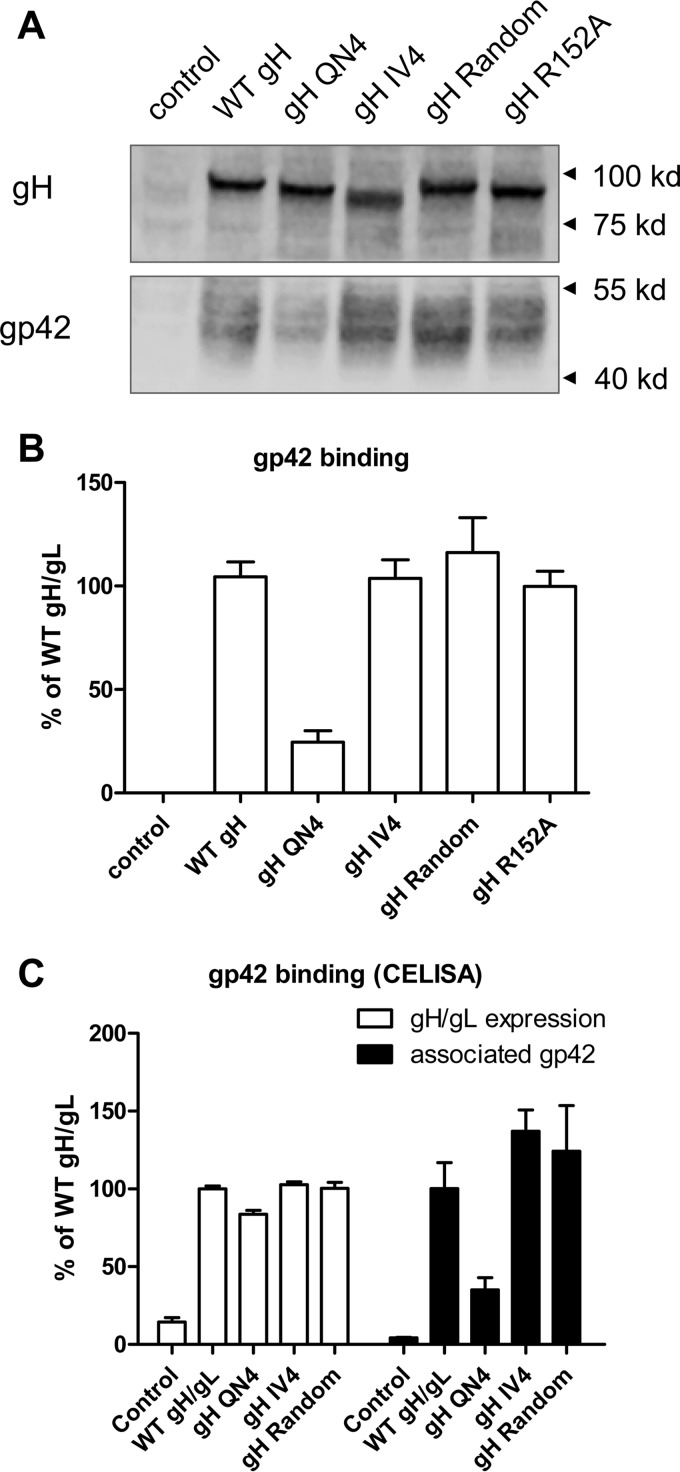
gH/gL CTD mutants with amino acid substitutions decreased association with gp42. (A) CHO-K1 cells seeded in six-well plates were transfected with control, WT gH/gL, or gH/gL mutants as indicated. A gp42 binding assay was performed to detect the Flag-tagged gp42 that bound to the transfected cells. Anti-gH was used as a loading control. (B) The relative level of gp42 binding was quantified and was normalized to the level of gH by using Image Studio software. The binding of WT gH/gL to gp42 was set at 100%. Data are means plus standard errors of the means for three independent experiments. (C) CHO-K1 cells seeded in six-well plates were transfected with control, WT gH/gL, or gH/gL mutants as indicated. Transfected cells were seeded in a 96-well plate in triplicate posttransfection. After 24 h, cells were washed twice with ice-cold PBS and incubated with soluble Flag-gp42 for 1 h at 4°C. The cells were then washed with ice-cold PBS four times, and the gH/gL surface expression and associated gp42 were determined by CELISA using antibodies E1D1 (gH/gL expression) and PB1112 (associated gp42).

Previous research on the alphaherpesvirus VZV showed that transient expression of gB and gH/gL of VZV is necessary and sufficient for inducing cell fusion in transfected cells. Mutating the last 8 amino acids (834 to 841) of VZV gH results in enhanced fusion. This role of the last 8 amino acids of VZV gH was dependent upon the physical length of the domain, not a specific sequence (21). Previous studies with HSV gH indicated that a serine-valine-proline (SVP) motif in the cytoplasmic tail may be important for HSV gH-mediated fusion (22). To examine if the EBV gH CTD amino acid composition is important for fusion activity, we generated additional EBV gH CTD mutants with a hydrophilic gH CTD (gH QN4, containing 4 repeats of hydrophilic residues glutamine and asparagine), hydrophobic gH CTD (gH IV4, containing 4 repeats of isoleucine and valine), or random amino acid (gH random) cytoplasmic tail (Fig. 3A). We tested each of the mutants for cell surface expression and fusion activity (Fig. 3) as well as gp42 binding ability (Fig. 4). We found that although all the gH CTD substitution mutants had WT levels of gH/gL cell surface expression (Fig. 3B), the fusion activity for both epithelial and Daudi B cells was decreased (Fig. 3C and D). Similar results were observed using a syncytium formation assay (Fig. 3E). This was also observed for the gH CTD mutant when the last 6 gH amino acids were replaced by a six-His tag (data not shown). These data indicate that in addition to the length, the amino acid composition of gH is also important for EBV gH function. Interestingly, unlike the truncation mutants, for which the B cell fusion activity corresponded to gp42 binding, there was little correlation of B cell fusion activity and gp42 binding for these gH CTD mutants that have hydrophilic, hydrophobic, or random amino acid composition in the gH cytoplasmic tail (Fig. 3D and 4). Although all of the three gH CTD substitution mutants had decreased B cell fusion activity, only gH CTD QN had decreased gp42 binding.

### Binding of gH conformational antibodies to the gH cytoplasmic tail mutants.

**FIG 5:**
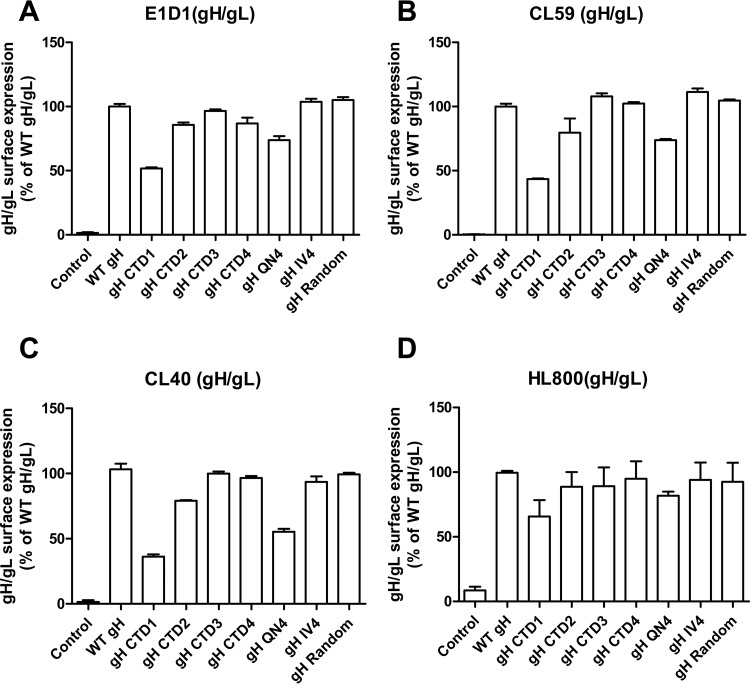
gH CTD mutants are recognized by gH/gL antibodies. CHO-K1 cells were transiently transfected with either control, WT gH/gL, or gH CTD mutants with gL. CELISA was performed with anti-gH/gL MAb E1D1 (A), CL59 (B), or CL40 (C) or polyclonal antibody HL800 (D).

Since our results with the gH CTD mutants suggested changes in the gH ectodomain conformation, we analyzed the cell surface expression of gH with three conformation-specific gH or gH/gL monoclonal antibodies (MAbs) (Fig. 5A to C) or a polyclonal gH/gL rabbit antibody (Fig. 5D) to determine if there were any differences in binding of gH/gL mutants to the different antibodies. As shown in Fig. 5, the different gH CTD mutants exhibited different levels of binding to the different antibodies. The MAbs CL40 and CL59 detect gH, and E1D1 recognizes an epitope formed by D-I and D-II of gH and gL (25, 26). CL59 recognizes an epitope within gH domain IV, including the flap region that comprises residues 500 to 629 (13, 26). The binding epitope for CL40 is not known (13). As shown in Fig. 5, compared to WT gH, the gH CTD1 mutant had a 50% decreased cell surface expression for all the antibodies. This indicates that the decrease is related to lower cell surface expression. For the other gH CTD mutants, a similar pattern of expression was observed for all the antibodies tested, indicating that there was no change in gH/gL conformation as probed with this small panel of gH/gL antibodies.

### gH CTD mutants binding to epithelial cells.

**FIG 6:**
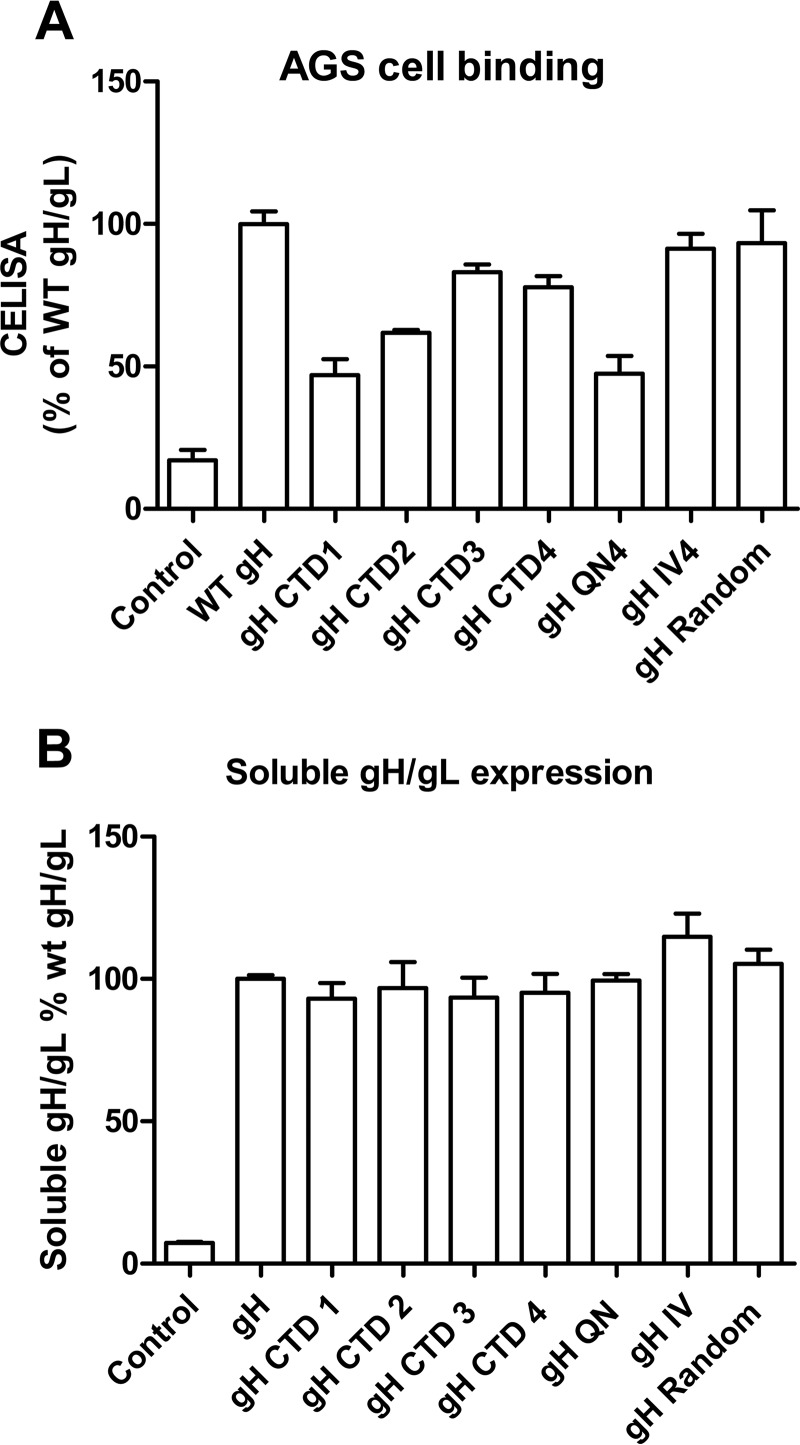
gH CTD mutants binding to AGS cells. CHO-K1 cells were transiently transfected with either control, WT gH/gL, or gH CTD mutants with gL. Soluble gH/gL and gH CTD mutants with gL were prepared using freeze-thawing methods and were used to overlay epithelial cells in 96-well plates in triplicate. After incubation for 4 h at 4°C, the binding in 96 wells was examined by CELISA using the E1D1 monoclonal antibody, which recognizes the gH/gL complex.

To investigate if reduced epithelial cell fusion observed with the gH CTD mutants was related to epithelial cell binding and by extension to the epithelial receptor or receptors, we performed a cell binding assay with solubilized gH/gL as described in Materials and Methods. As shown in Fig. 6, the gH CTD1, gH CTD2, and gH QN4 mutants had decreased epithelial cell binding consistent with the lower epithelial fusion that we observed. However, although the gH CTD IV mutant and the gH CTD random amino acid mutant had decreased fusion activity, there was no change in epithelial cell binding.

### The cytoplasmic tails of EBV gB and gH are not interchangeable but may interact.

**FIG 7:**
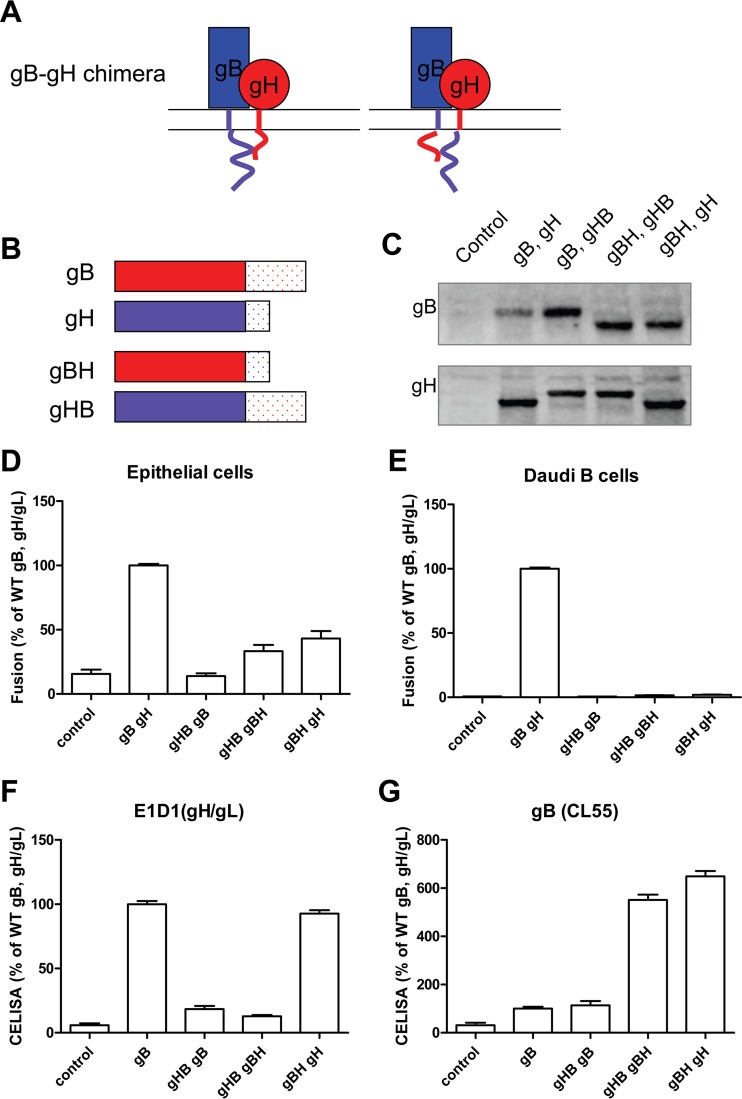
CTDs of EBV gH and gB are not interchangeable. (A and B) Schematic of gB-gH tail exchange chimeric mutants. (C) Expression of gB-gH tail mutants in transfected CHO-K1 cells was examined using Western blotting. (D and E) CHO-K1 cells were transiently transfected with T7 luciferase plasmid alone with vector plasmid (control), T7 luciferase plasmid, or EBV gL, gB, gH, or gB-gH tail exchange chimera mutants as indicated in the absence (D) or presence (E) of gp42. Twenty-four hours posttransfection, transfected CHO-K1 cells were overlaid with epithelial (D) or Daudi B (E) cells expressing T7 polymerase, and luciferase activity was monitored 24 h after overlay and normalized to cells with the WT gH/gL value set to 100%. The data are the mean plus standard error of the mean from three independent experiments. (F and G) Cell surface expression of gB-gH detected by CELISA using anti-gH/gL monoclonal antibody E1D1 (F) or anti-gB monoclonal antibody CL55 (G).

**FIG 8:**
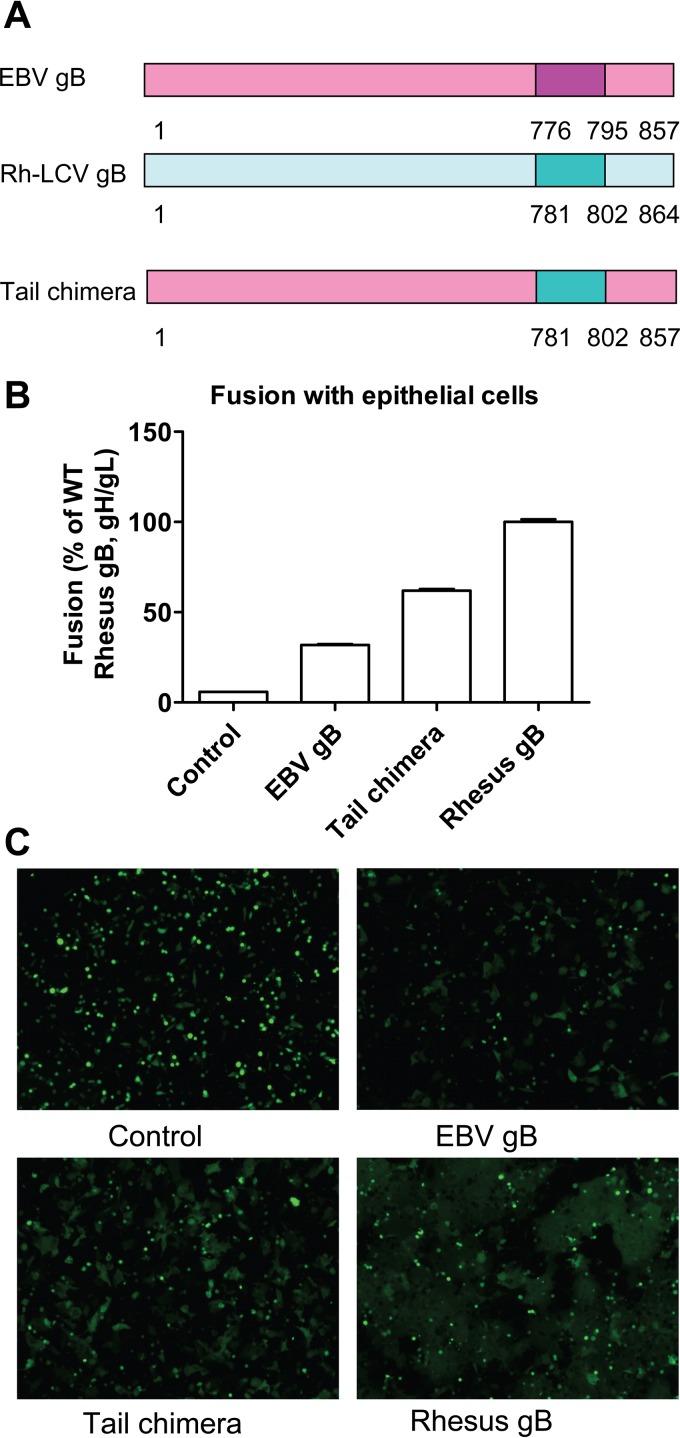
The CTDs of EBV gH and gB are not interchangeable. (A) Schematic of EBV/Rh gB tail chimera. CHO-K1 cells were transiently transfected with T7 luciferase plasmid alone with vector plasmid (control), T7 luciferase plasmid and rhLCV gH/gL plus EBV gB, EBV/Rh gB chimera, or rh LCV gB as indicated. (B) Twenty-four hours posttransfection, transfected CHO-K1 cells were overlaid with HEK 293 cells expressing T7 polymerase, and luciferase activity was monitored 24 h after overlay and normalized to cells with the WT gH/gL value set to 100%. The data are the mean plus standard error of the mean from three independent experiments. (C) CHO-K1 cells were transfected with green fluorescent protein plasmid alone with vector plasmid (control), green fluorescent protein plasmid and rhLCV gH/gL plus EBV gB, EBV/Rh gB chimera, or rh LCV gB as indicated and overlaid with HEK 293 cells. Syncytium formation was visualized and captured with a Leica fluorescence microscope.

Previously it was shown that the cytoplasmic tails of both EBV gB and gH are essential components for the cell-cell fusion mechanism, and a recent study of HSV gH/gL indicated that the gH/gL cytoplasmic tail may act as “wedge” to release the gB cytodomain “clamp” (23, 27). Thus, we hypothesized that gH and gB cytoplasmic tails may interact with each other, and as a result, the CTDs of gH and gB may be interchangeable. To test this hypothesis, we generated chimeric gB and gH by exchanging their cytoplasmic tails (Fig. 7A). We transfected CHO-K1 cells with control, WT gH/gL, gB, or the chimeric gH or gB mutants as indicated. The total amount of the protein was determined by Western blotting. Compared to the WT gB, the chimeric gB with the cytoplasmic tail of gH (gBH, Fig. 7C, lanes 4 and 5) had a lower apparent molecular weight. In contrast, the chimeric gH with the cytoplasmic tail of gB (gHB, Fig. 7C, lanes 3 and 4) had a higher apparent molecular weight than WT gH. These changes in apparent molecular weights were expected due to the difference in amino acid length of the gH and gB CTDs. To examine if the gH-gB chimeras mediate fusion like WT gH and gB, CHO-K1 cells were transfected with T7 luciferase, gL along with control, or different combinations of gH, gB, or gH and the gB chimeras as indicated (Fig. 7D). We performed similar transfections in the presence of gp42 (Fig. 7E). For epithelial cell fusion, the combination of gHB and gB did not mediate fusion (Fig. 7D). Interestingly, the combination of gHB/gBH or gBH/gH resulted in a small increase in fusion activity compared to the control only (Fig. 7D), likely due to the increased cell surface expression of gBH (Fig. 7G). For Daudi B cell fusion, except for the WT gH and gB, neither of the chimera combinations had fusion activity (Fig. 7E). In addition to the fusion activity, we also examined the cell surface expression of the chimeras (Fig. 7F and G). The data showed that gH with a gB CTD was not expressed on the cell surface while gB with the shorter gH CTD had higher cell surface expression, similar to the data shown before, indicating that gB with a shorter CTD had increased cell surface expression, likely due to the deletion of gB CTD residues important for the regulation of cellular localization (23, 28). Since gH with the long gB tail was not expressed well on the cell surface of CHO cells and as a result is defective in cell fusion, we decided to use an alternative method to test the hypothesis that there is cross talk between the gH CTD and gB CTD. Our previous results found that EBV gB does not complement gB encoded by the EBV-related rhesus lymphocryptovirus (rhLCV) in fusion function due to a species-specific dependence between gB and gH/gL (29). To determine the importance of the tail domain in any potential interaction of the gB and gH CTDs, we constructed a chimeric rhLCV and EBV gB focusing on the CTD. Analysis of EBV/Rh gB chimeric proteins from this earlier study showed that insertion of rhLCV gB from residues 456 to 807 restored fusion function of EBV gB with rhLCV gH/gL, suggesting that this region of gB is important for interaction with gH/gL (29). To determine if the CTD may be important for this observation, we generated an EBV/Rh gB chimera with the EBV gB CTD (aa 776 to 795) replaced with the corresponding region of the rhLCV gB tail (aa 781 to 802) and tested fusion activity with this chimeric protein (Fig. 8). Interestingly, this mutant was partially restored for fusion function with rhLCV gH/gL, indicating a potential direct interaction between the EBV gB CTD and gH/gL CTD within a region of the gB CTD proximal to the membrane.

In summary, our current results indicate that the first 2 amino acids after the transmembrane domain are important for the stability of gH, gH transport to the cell surface, and retention in the plasma membrane while the first 4 amino acids after the transmembrane domain are important for fusion activity and the ability of gH to bind to gp42 and to epithelial cells.

## DISCUSSION

The crystal structures of ectodomains of EBV gH/gL and EBV gB have been previously solved (4, 5); however, there is no information regarding the structure of the TMD or CTD of these two glycoproteins. Past studies examining the CTDs of herpesvirus-encoded gH and gB family members have shown that the CTDs play an important role in fusion regulation (16, 22, 30). Alanine substitution of conserved residues in the cytoplasmic tail of HSV gB can either enhance or abolish cell fusion activity and viral entry (30). These data are similar to our findings that the EBV gB CTD regulates fusion activity, as shown by studies of a comprehensive library of mutants with truncations of EBV gB CTD (23). Previous studies also showed that a region from amino acids 802 to 816 is necessary for productive membrane fusion, while amino acids 817 to 841 comprise a domain that negatively regulates membrane fusion (31).

Interestingly, although both positive and negative roles of the gH CTD have been reported in different herpesviruses, there is no reported example of a particular herpesvirus gH CTD having both functions. This may be related to the longer length of the gB CTD compared to the gH CTD. For example, the EBV gH CTD has only 8 amino acids, while the EBV gB CTD has 104 amino acids. This added length may have allowed for different functional domains to evolve to regulate fusion function. In addition, since gB is the viral fusogen, it may require precise regulation that is afforded by additional functional domains within the gB CTD. Similar regulation has also been reported in other virus fusion proteins, such as the Nipah virus fusion protein (NiV-F) and other paramyxoviruses. In NiV-F, there is a tribasic KKR motif in the membrane-adjacent region at the NiV-F cytoplasmic tail. Mutation of the first K to an A increases fusion 5.5-fold, while the second K-to-A and third R-to-A mutations decreased fusion 3- to 5-fold (32). For other paramyxoviruses, alanine scanning mutagenesis of a membrane-proximal 7-residue external region (MRER) indicates that this MRER has both positive and negative regulatory roles in fusion, as both hyperfusogenic and hypofusogenic mutations were found (33).

Our current studies were designed to investigate the role of the EBV CTD in fusion. To do this, we generated a series of EBV gH CTD truncation mutants. We used both a luciferase-based cell-cell fusion assay and a syncytium formation assay to systematically investigate the dependence on the length of the gH CTD for fusion, since these two assays gave different results previously in the analysis of the CTD of HSV gH (17). Unlike HSV gH, removal of the entire CTD did not affect the cell surface expression determined by fluorescence-activated cell sorting (FACS) (27). Removal of the entire EBV gH CTD resulted in an approximately 50% reduction in cell surface expression determined by CELISA. The discrepancy between the HSV and EBV data is likely due to different properties of the gH CTD but also may be due to the methods used for detection. The deletion of the EBV gH C-terminal 4 residues (MFFL) had no effect on fusion activity and cell surface expression. Further deletion of the amino acids next to the MFFL motif led to a drastic decrease of fusion activity for both epithelial cells and Daudi B cells. This is consistent with the results from an HSV gH CTD study showing that the first eight residues of the 14-residue gH CTD were sufficient to mediate fusion at WT levels (22, 27, 34). Further deletion of the HSV gH cytoplasmic tail resulted in reduced fusion activity, and the decreased level was proportionate to the CTD length (27). Interestingly, the gH CTD1 also has a dominant negative effect on WT gH when tested for fusion function (data not shown), potentially as a result of maintaining interaction with gB but being nonfunctional in triggering gB activation and subsequent fusion.

The decreased fusion activity of gH mutants with Daudi B cells is often accompanied by decreased gp42 binding (11, 12). Two forms of gp42 are generated when gp42 is expressed in cells (35). There is a full-length membrane-bound form and a soluble form generated by proteolytic cleavage in the endoplasmic reticulum (ER) with cleavage occurring around amino acids 40, 41, and 42 (35). A membrane-bound form of gp42 lacking the predicted cleavage site (residues 37 to 41) blocks B cell fusion (36). gp42 has to be cleaved to bind gH/gL to activate entry into B cells by interaction with human leukocyte antigen (HLA) class II (36). Thus, the ability of gH/gL to bind to soluble gp42 can be used as an indication of the conformation of the gH/gL ectodomain. Using a gp42 binding assay, we determined that the gH CTD truncation mutants had decreased fusion activity (Fig. 1D) and decreased gp42 binding (Fig. 2), which correlated with Daudi B cell fusion activity (Fig. 1D and 2). Using a soluble gH binding assay that we developed for monitoring binding to the epithelial cell receptor (11, 12), we found that the ability of the gH CTD1 and gH CTD2 mutants to bind to epithelial cells was also decreased, resulting in the loss of epithelial cell fusion (Fig. 1 and 6).

Taken together, our data indicate that deletion of the 4 amino acids at the N-terminal portion of the gH CTD is critical for fusion function and may affect the gH ectodomain conformation. We also used a panel of conformational antibodies specific to the ectodomain of gH or gH/gL to confirm our hypothesis. However, no significant conformational differences were observed as shown in Fig. 5 with this small panel of gH/gL antibodies, potentially indicating that there is no large conformational changes in gH/gL. There is another possibility that the cytoplasmic tail of the EBV gH/gL could regulate the endosomal trafficking or glycosylation. The contribution of the cytoplasmic tail of a fusion protein in regulating endosomal trafficking has been shown for the Nipah virus fusion protein (37).

The last 8 amino acids (aa 834 to 841) located at the VZV gH CTD are linked to the regulation of gB- and gH/gL-mediated cell fusion as corroborated by increased cell fusion *in vitro* when aa 834 to 841 of VZV gH are truncated. This gH cytoplasmic domain regulation is dependent on the physical presence of amino acids 834 to 841, since replacement of aa 834 to 841 with V5, cMyc, or hydrophobic or hydrophilic sequences did not affect fusion while stepwise deletions of residues 834 to 841 caused incremental increases in cell fusion (21). To verify if the regulation of the EBV gH CTD is dependent on the length but not specific biochemical properties of the domain, we changed the characteristics of the EBV gH CTD to be hydrophobic (IV4) or hydrophilic (QN4) or to contain a random amino acid sequence. Unlike the VZV gH tail, the replacement of the EBV gH cytoplasmic tail resulted in the reduction of fusion in both epithelial cell and Daudi B cell fusions for all the substitution mutants tested (Fig. 3), indicating a specific requirement for the EBV gH CTD amino acid composition. This is consistent with the HSV-1 gH study showing that the replacement of the gH TMD or CTD with analogous sequences from other transmembrane proteins resulted in chimeric molecules that were unable to mediate fusion and were nonfunctional or greatly impaired in their ability to mediate virus entry (16). Although all the substitution mutants had defective fusion activity, only gH QN4 had decreased gp42 binding and epithelial cell binding (Fig. 5 and 6), indicating that there may be another potential mechanism that regulates fusion through the gH cytoplasmic tail.

Interestingly, the VZV gH CTD and HSV gH CTD had been proposed to function as a potential gatekeeper, with the length of the CTD controlling access to functional domains within neighboring proteins such as gB or acting as a “wedge” to release a gB cytoplasmic domain “clamp” and enable gB activation, respectively (21, 27). Moreover, for HSV gH, the removal of the entire gH CTD abolished fusion with all gB constructs, including hyperfusogenic mutants, indicating the importance of the gH CTD (27). For VSV gH, the hyperfusogenic phenotype of gB mutants Y881F and Y881/920F is also dependent upon the absence of amino acids 834 to 841 of gH, suggesting that the cytoplasmic domains of gB and gH might function together in regulating cell fusion (21). We also tested the fusion activity of our gH CTD mutants in the presence or absence of gB843, a hyperfusogenic mutant that we previously identified (data not shown) (23, 24). For WT gH, the gB843 mutant increased fusion activity 4-fold, but for the gH mutant with a fully truncated CTD, only a 2-fold increase was observed with the gB843 mutant. It is possible that there are direct or indirect interactions of the gB CTD and gH CTD. Previously, studies with HSV demonstrated that there was no interaction between a recombinant gB CTD expressed in *Escherichia coli* and a synthetic peptide encompassing the gH cytoplasmic tail CTD (17). This may suggest that the interaction is transient or may require the transmembrane region.

To test the hypothesis that the presence of the gB CTD and gH CTD is sufficient to support fusion activity, we generated gB and gH chimeras (gHB and gBH) by exchanging their respective CTDs. However, the gHB and gBH did not mediate fusion, likely due to the lack of cell surface expression of gHB. One interesting result that we observed was that the combination of gHB and gBH produced slightly more fusion activity than the combination of gHB and gB. This is likely due to higher cell surface expression of the gBH chimera than of WT gB. These data further confirmed that both length and amino acid composition are important for gH and gB tail function. Previous data from our lab indicated that EBV gB does not complement rhLCV gB due to a species-specific dependence between gB and gH/gL. Interestingly, when we replaced the tail domain of EBV gB with the corresponding rhLCV gB, the EBV/Rh gB tail chimera partially restored fusion function with rhLCV gH/gL, indicating a potential interaction of the gB CTD with the gH/gL CTD.

To summarize, the role of the EBV gH CTD in membrane fusion was investigated by generating a panel of deletion and amino acid substitution mutants within the gH CTD. We found that the fusion function of the EBV gH CTD depends on both the length and specific sequence or properties of the amino acids in the gH CTD. The first four of the 8 amino acids are essential for the function of the gH CTD. Interestingly, by exploring gp42 binding and epithelial cell binding of our panel of gH CTD mutants, we found that the gH CTD may regulate the ectodomain conformation, resulting in decreased gp42 and epithelial cell binding. In the future, we will further determine how the CTDs of gH/gL and gB interact with each other and regulate fusion activity.

## MATERIALS AND METHODS

### Cell culture.

Chinese hamster ovary cells (CHO-K1) cells were grown in Ham’s F-12 medium (Corning) containing 10% heat-inactivated fetal bovine serum (FBS) complex (Corning) and 1% penicillin-streptomycin (100 U penicillin/ml and 100 µg streptomycin/ml; Sigma). The Daudi 29 cell line (used for B cell fusion) and human embryonic kidney 293 (HEK 293) cells (used for epithelial cell fusion) stably expressing T7 RNA polymerase (38, 39) were grown in RPMI 1640 medium (Corning) with 900 µg/ml G418 (Sigma) and Dulbecco modified Eagle medium (DMEM) with 100 µg/ml zeocin (Invitrogen), respectively, containing 10% heat-inactivated FBS and 1% penicillin-streptomycin.

### Construction.

Mutations of the gH C-terminal domain (CTD) (gH CTD1, gH CTD2, gH CTD3, gH CTD4, gH QN4, gH IV4, and gH random) were generated using a QuikChange site-directed mutagenesis kit (Stratagene). The primers used are shown in Table 1. The construction of a gH and gB chimera (gHB) as well as an EBV/Rh gB chimera was done using overlapping PCR with the primers listed in Table 1. The construction of gB and gH chimera gBH was done using a gBlocks gene fragment from Integrated DNA Technologies (IDT). Sequencing was done to confirm the presence of the mutations and the absence of second-site mutations.

**TABLE 1:** Sequences of primers for mutants

Primer no.	Primer name	Sequence
1	EBV gH CTD1 F	5′-ATCTTTCTGGTTTAGAAGATTGTTATG-3′
2	EBV gH CTD1 R	5′-CATAACAATCTTCTAAACCAGAAAGAT-3′
3	EBV gH CTD2 F	5′-CTGGTTCACAAGTAGGTTATGTTTTTC-3′
4	EBV gH CTD2 R	5′-GAAAAACATAACCTACTTGTGAACCAG-3′
5	EBV gH CTD3 F	5′-CACAAGATTGTTTAGTTTTTCCTTTAG-3′
6	EBV gH CTD3 R	5′-CTAAAGGAAAAACTAAACAATCTTGTG-3′
7	EBV gH CTD4 F	5′-ATTGTTATGTTTTAGCTTTAGATGCGC-3′
8	EBV gH CTD4 R	5′-GCGCATCTAAAGCTAAAACATAACAAT-3′
9	gH IV4 F	5′-CTGGGTATCTTTCTGGTTATCGTTATCGTTATCGTTATCGTTTAGATGCGCAGCAGGTAA-3′
10	gH IV4 R	5′-TTACCTGCTGCGCATCTAAACGATAACGATAACGATAACGATAACCAGAAAGATACCCAG-3′
11	gH QN4 F	5′-CTGGGTATCTTTCTGGTTCAGAACCAGAATCAGAATCAGAATTAGATGCGCAGCAGGTAA-3′
12	gH QN4 R	5′-TTACCTGCTGCGCATCTAAACGATAACGATAACGATAACGATAACCAGAAAGATACCCAG-3′
13	gH Random F	5′-CTGGGTATCTTTCTGGTTATCGTTACGTTATCGTTATCGTTTAGATGCGCAGCAGGTAA-3′
14	gH Random R	5′-TTACCTGCTGCGCATCTAAACGATAACGATAACGTAACGATAACCAGAAAGATACCCAG-3′
15	gH NheI F	5′-TAGAGATTGGGGAGGTTTTGC-3′
16	gH-gB chimera F	5′-GCTCTGGGTATCTTTCTGGTTAGGCGCACGCGCCAGATGTCG-3′
17	gH-gB chimera R	5′-CGACATCTGGCGCGTGCGCCTAACCAGAAAGATACCCAGAGC-3′
18	gB tail BglII R	5′-TGTGAGATCTTTAAAACTCAGTCTCTGCCTC-3′
19	10 EBV 287 XhoI F	5′-AAGCTCGAGAACAGGACAGCC-3′
20	EBV/Rh gB tail chimera F	5′-TGGGAACGGTGGAGCTGCCGTGGTAAGCTCCGTCAG-3′
21	EBV/Rh gB tail chimera R	5′-CTGACGGAGCTTACCACGGCAGCTCCACCGTTCCCA-3′
22	EBV622-PstI R	5′-GGTCTGCAGGGTGGCAATGCCGTC-3′

### Transfection.

CHO-K1 cells, grown to approximately 80% confluence, were transiently transfected with plasmids expressing the mutants and other essential glycoproteins for fusion, including gB (0.8 µg), gH (0.5 µg), gL (0.5 µg), and gp42 (0.8 µg), and a luciferase reporter plasmid with a T7 promoter (0.8 µg) by using Lipofectamine 2000 transfection reagent (Invitrogen) in Opti-MEM (Gibco-Life Technologies) as previously described (11). Equal amounts of glycoprotein mutant DNA were used in each experiment.

### Fusion assay.

The virus-free cell-based fusion assay was performed as described previously (11). Briefly, effector CHO-K1 cells were transfected as described above. Twenty-four hours posttransfection, the cells were detached, counted, and mixed 1:1 with target cells (Daudi 29 B cells or HEK 293 cells, 0.25 × 10^6^ per sample) into a 24-well plate in 1 ml Ham’s F-12 medium with 10% heat-inactivated FBS. Twenty-four hours later, the cells were washed once with phosphate-buffered saline (PBS) and lysed with 100 µl of passive lysis buffer (Promega). Luciferase was quantified in duplicate by transferring 20 µl of lysed cells to a 96-well plate and adding 100 µl of luciferase assay reagent (Promega), and luminescence was measured on a PerkinElmer Victor plate reader.

### CELISA.

The expression in the plasma membrane of the various mutants was determined by cell enzyme-linked immunosorbent assay (CELISA) as described in previous reports (11). CHO-K1 cells were transfected with various glycoproteins and mutants. Transfected cells were split for use in a fusion assay (above) and CELISA. Twenty-four hours posttransfection, 4 × 10^4^ cells/well were transferred to a 96-well plate and incubated for another 24 h. The expression of each glycoprotein (including mutants) was evaluated using conformation-specific antibody E1D1 for gH/gL or CL55 for gB. After incubation with primary antibody for 30 min and fixation with 2% formaldehyde and 0.2% glutaraldehyde in PBS for 15 min, an anti-mouse biotin-labeled secondary antibody was added at 1:500 dilution and incubated for 30 min. After washing, streptavidin-labeled horseradish peroxidase (1:20,000) was further incubated with the fixed cells for 30 min. Peroxidase substrate was added, and the amount of cell surface staining was determined by measurement at 380 nm with a PerkinElmer Victor plate reader.

### Soluble gH binding assays.

CHO-K1 cells were transfected with gH/gL or gH CTD mutants as described above. Twenty-four hours later, 2 × 10^6^ cells were collected, resuspended in 1 ml medium, subjected to 3 cycles of freeze-thaw in liquid nitrogen, and sonicated for 10 s. Insoluble cellular debris was removed by centrifugation at 1,500 × *g* for 5 min. AGS cells were overlaid with clarified supernatants (100 µl) in a 96-well dish in triplicate. After incubation for 4 h at 4°C, gH/gL binding was examined using CELISA as described below.

### gp42 binding assay.

CHO-K1 cells, grown to 80% confluence, were transfected with wild-type (WT) soluble Flag-gp42 (40) in 6-well plates. Other 6-well plates were similarly transfected as described above with control, gH/gL, or gH CTD mutants using Lipofectamine 2000. Twenty-four hours posttransfection, the supernatant of the cells transfected with soluble Flag-gp42 was collected and centrifuged to collect soluble gp42 contained in the medium supernatant. Cells transfected with control, gH/gL, or the gH CTD mutants were washed twice with ice-cold PBS and incubated with Flag-gp42 for 1 h at 4°C. The cells were then washed with ice-cold PBS four times and lysed with 200 µl of SDS sample buffer. Proteins were separated on Bio-Rad 10% mini-Protean TGX gels after boiling for 10 min under reducing conditions. Western blot analyses were performed using a polyclonal anti-Flag antibody (F7425; Sigma) at 1:1,000 or polyclonal anti-gH/gL antibodies at 1:100 (10). To determine gp42 binding using CELISA, transfected cells were seeded in a 96-well plate in triplicate 24 h posttransfection. Following 24 h of growth, the transfected cells were washed twice with ice-cold PBS and incubated with soluble Flag-gp42 for 1 h at 4°C. The cells were then washed with ice-cold PBS four times, and the gH/gL surface expression and associated gp42 were determined by CELISA.

### SDS-PAGE migration assay.

CHO-K1 cells were transfected with control or the plasmids as indicated in the figure legends. After 24 h, transfected cells in 6-well plates were collected and lysed in 200 µl lysis buffer (20 mM Tris-HCl, pH 7.4, 100 mM NaCl, 1 mM EDTA, 5 mM MgCl_2_, 1% Triton X-100, and Calbiochem 1× protease inhibitor cocktail set I). The cell lysates were cleared of debris by centrifugation, and 100 µl of the lysates was mixed with 100 µl 2× SDS loading buffer (60 mM Tris-Cl, pH 6.8, 0.2% SDS, 25% glycerol, 0.01% bromophenol blue). The samples were loaded onto a Bio-Rad 7.5% mini-Protean TGX gel for Western blotting. After electrophoresis, proteins were transferred to nitrocellulose membranes (Schleicher & Schuell, Keene, NH). The blots were blocked with 5% nonfat dry milk in Tris-buffered saline (TBS) buffer (20 mM Tris-HCl, pH 7.6, 137 mM NaCl) for 2 h at room temperature (RT). The blots were washed with TBS and incubated with primary antibodies (anti-gB or anti-gH/gL) overnight at 4°C. Anti-rabbit IRDye800 or anti-mouse IRDye680 secondary antibodies (Li-Cor Bioscience, Lincoln, NE) were added to the membranes at a dilution ratio of 1:10,000, and incubation was continued for 1 h at RT. Protein bands on the membrane were visualized with the Odyssey Fc Western blotting imager using Image Studio version 2.0 (Li-Cor Bioscience, Lincoln, NE).
